# The effects of a computerized clinical decision aid on clinical decision-making and guideline implementation in psychosis care

**DOI:** 10.1192/j.eurpsy.2022.479

**Published:** 2022-09-01

**Authors:** S. Castelein, L.O. Roebroek, A. Boonstra, W. Veling, F. Jörg, B. Sportel, J. Bruins, P.A.E.G. Delespaul

**Affiliations:** 1 Lentis Psychiatric Institute, Lentis Research, Groningen, Netherlands; 2 University of Groningen, Clinical Psychology, Groningen, Netherlands; 3 University of Groningen, Faculty Of Economics And Business, Groningen, Netherlands; 4 University Medical Center Groningen, University Center For Psychiatry, Groningen, Netherlands; 5 GGz Drenthe, Psychosis Department, Assen, Netherlands; 6 Maastricht University, School For Mental Health And Neurosciences, Maastricht, Netherlands; 7 Mondriaan, Research, Heerlen, Netherlands

## Abstract

**Introduction:**

Clinicians in mental healthcare have few objective tools to identify and analyse their patient’s care needs. Clinical decision aids are tools that can support this process.

**Objectives:**

This study examines whether 1) clinicians working with a clinical decision aid (TREAT) discuss more of their patient’s care needs compared to usual treatment, and 2) agree on more evidence-based treatment decisions.

**Methods:**

Clinicians participated in consultations (n=166) with patients diagnosed with psychotic disorders from four Dutch mental healthcare institutions. Primary outcomes were measured with the modified Clinical Decision-making in Routine Care questionnaire and combined with psychiatric, physical and social wellbeing related care needs. A multilevel analysis compared discussed care needs and evidence-based treatment decisions between treatment as usual (TAU) before, TAU after and the TREAT-condition.

**Results:**

First, a significant increase in discussed care needs for TREAT compared to both TAU conditions (b = 20.2, SE = 5.2, p = 0.00 and b = 15.8, SE = 5.4, p = 0.01) was found. Next, a significant increase in evidence-based treatments decisions for care needs was observed for TREAT compared to both TAU conditions (b = 16.7, SE = 4.8, p = 0.00 and b = 16.0, SE = 5.1, p = 0.01).

**Conclusions:**

TREAT improved the discussion about physical health issues and social wellbeing related topics. It also increased evidence-based treatment decisions for care needs which are sometimes overlooked and difficult to treat. Our findings suggest that TREAT makes sense of ROM data and improves guideline-informed care.

**Disclosure:**

No significant relationships.

